# Bilateral Patterns of Repetitive Movements in 6- to 12-Month-Old Infants with Autism Spectrum Disorders

**DOI:** 10.3389/fpsyg.2017.01168

**Published:** 2017-07-11

**Authors:** Giulia Purpura, Valeria Costanzo, Natasha Chericoni, Maria Puopolo, Maria Luisa Scattoni, Filippo Muratori, Fabio Apicella

**Affiliations:** ^1^Department of Developmental Neuroscience, IRCCS Stella Maris Foundation Pisa, Italy; ^2^Department of Cell Biology and Neurosciences, Istituto Superiore di Sanità Rome, Italy; ^3^Research Coordination and Support Service, Istituto Superiore di Sanità Rome, Italy; ^4^Department of Clinical and Experimental Medicine, University of Pisa Pisa, Italy

**Keywords:** Autism Spectrum Disorders, repetitive movements, home videos, bilateral patterns, early signs of autism

## Abstract

**Aim:** Some patterns of repetitive movements and their frequency have been proved to distinguish infants with Autism Spectrum Disorders (ASD) from infants with Typical Development (TD) and Developmental Delay (DD) from 12 months of life on. The purpose of this study is to investigate if a specific repertoire of repetitive movements is present earlier in life, and if their higher rate and duration could differentiate infants with ASD from infants with DD and TD aged between 6 and 12 months.

**Method:** We conducted a retrospective analysis of video-clips taken from home videos to compare the frequency and the duration of Repetitive Movement Episodes (RMEs) in a sample of 30 children equally distributed among the three groups.

**Results:** Significantly higher total scores in bilateral RMEs with arms, hands, fingers, and lower limbs were found to distinguish ASD infants from both DD and TD infants, with a satisfactory diagnostic efficiency. No significant difference was found between the distributions of unilateral RMEs between ASD and DD/TD.

**Interpretation:** Results indicate the presence at this age of an ASD-specific pattern of bilateral repetitive movements. We hypothesize a continuum between this pattern and the lack of variability in finalized and communicative movements and gestures observed in children with ASD during the second year of life.

## Introduction

The presence of repetitive movements, described as the repetition of the same movement multiple times, is considered a necessary step for the development of voluntary purposeful movements and seems to have an adaptive role during limited temporal windows of psychomotor development (Thelen, 1979). Despite the fact that a certain motor repetitiveness in early infancy is considered a requirement for typical motor development, an increased frequency of repetitive movements has been widely described in abnormal neurodevelopmental trajectories, reflecting a continuum that extends from typical to atypical development (Leekam et al., 2007). Several studies also documented broad patterns of repetitive movements in infants with neurodevelopmental disorders (i.e., Morgan et al., 2008), suggesting that a higher rate of repetitiveness could affect the quality of the infant’s repertoire of motor behaviors. The term repertoire is used to describe the full range of motor behavior that an infant is provided with. In typical development (TD), the natural repertoire of early spontaneous motor activity, which is also an expression of spontaneous neural activity (Einspieler and Prechtl, 2005), is able to provide the newborn’s brain with the appropriate opportunities for psychomotor learning by direct experience. The term “psycho-motor” has been used to highlight the close relationship between motor development and changes in a child’s cognitive, emotional, and social capacities (Cioni and Sgandurra, 2013). Psychomotor learning benefits from a good variation (defined as a broad repertoire of behaviors for a specific motor function) and a good variability (that is the capacity to select from a broad repertoire the motor strategy that fits the situation best). Together, variation and variability constitute the child’s adjustment to the environment and facilitate functional learning (Hadders-Algra, 2010). During typical motor development, motor variation seems to precede and lay the foundation for the ability to adapt the acquired repertoire of movements to specific functions and to expand variability. Motor variation and variability could be considered an indication of typical motor development (Dusing and Harbourne, 2010) and, consequently, a hallmark of typical spontaneous activity in the nervous system. Particularly, motor variation, expressed through a broad repertoire of different types of movements, seems to be a necessary prerequisite for variability, expressed through the functional and adaptive use of a motor behavior.

If, on one hand, the concept of motor variation is considered as an indicator of typical motor development, on the other hand a certain degree of motor repetitiveness is also considered a milestone for the development of more functional motor actions. The phenomenology and terminology of repetitive movements has been the subject of debate (Hedderly, 2016). From 6 weeks of age, the infant repeatedly produces gross movements, such as kicking, wiggling-oscillating and swiping (Prechtl and Hopkins, 1986). These motor activities with arms, hands, and lower limbs continue until 12 months of life, progressively diminishing in intensity and frequency and giving way to voluntary movements of locomotion (Thelen, 1979). In the same period the infant strikes and shakes objects, experiencing so-called object banging, in order to discover the physical characteristics of the objects and to train praxis abilities. This behavior takes the form of an action that is pre-adapted for tool use (Kahrs et al., 2013).

To distinguish between typical and atypical repetitive movements, the criteria that should be adopted are the child’s age and the quality of performance. The persistence of repetitive and stereotyped movements during toddlerhood is considered, together with restricted interests and activities, one of the core symptoms of Autism Spectrum Disorder (ASD) (American Psychiatric Association, 2013).

The major issue regarding the analysis of repetitive and stereotyped movements is their presence both in children with Developmental Disability (DD) (Mahone et al., 2004; Morgan et al., 2008) and with TD (Thelen, 1979; Leekam et al., 2007), raising the question whether they can be considered a sensitive marker of ASD. Attempts to answer this question were made by two parallel studies on siblings and on clinical populations of toddlers during their second year of life. Loh et al. (2007) carried out a prospective study on repetitive movements using Thelen’s taxonomy (Thelen, 1979) as a framework, in siblings subsequently diagnosed with ASD, undiagnosed siblings and siblings of typically developing infants. In this study several types of repetitive movements were reported in all three groups at 12 months of age; nevertheless at this age “Arm Wave” occurred more often in infants later diagnosed with ASD compared only to TD infants, while at 18 months “Arm Waving” became specific to the ASD group, compared to the other two groups. Morgan et al. (2008) compared videotaped sessions of children with ASD, DD, and TD, within the age range 18–24 months, and found a significantly higher rate and a larger inventory (described as an index of the motor repertoire during a single observation) of repetitive movements both with and without objects in the ASD group compared to the other two groups. This study suggests that not only a higher frequency but also a broader repertoire of repetitive movements is specific to the ASD developmental pathway. Furthermore, significant correlations were found between repetitive movements and measures of social communication, supporting the idea that the frequency of repetitive movements in children under 24 months of age has a close relationship with core elements of ASD.

Kim and Lord (2010) found stable diagnostic differences between toddlers with ASD, toddlers with non-spectrum disorders and TD toddlers, in the prevalence and severity of restricted and repetitive patterns of behaviors, measured during the ADOS. Differences were clear from 12 months of age, suggesting that the analysis of repetitive behaviors could play a key role in the early diagnosis of ASD.

Damiano et al. (2013) suggests that the higher rate and number of different types of repetitive movements in high-risk infant siblings compared to low-risk infant siblings may represent a broader behavioral phenotype that is common in high-risk populations, although it does not always portend a later diagnosis of ASD. On the contrary, Elison et al. (2014) and Wolff et al. (2014) found that the rate of repetitive movements distinguishes high-risk (HR) infants with a later diagnosis of ASD (HR-ASD) not only from TD infants but also from undiagnosed high-risk siblings (HR-no ASD).

Summarizing, the literature suggests that the frequency of repetitive movements should be considered, from 12 months onward, as a red flag for neurodevelopmental disorders such as ASD. On the other hand, evidence of the presence of motor abnormalities in ASD before the first birthday is lacking (Baranek, 1999; Werner and Dawson, 2005), controversial (Osterling et al., 2002) or primarily concerns gross or fine motor development in general. For example, Ozonoff et al. (2008) in their retrospective analysis of infant home videos, found that infants subsequently diagnosed with ASD showed a delay in the acquisition of gross motor milestones during the first year of life. Additionally, Libertus et al. (2014) found an immaturity in fine motor and grasping skills in infants at risk for ASD, both with or without an ASD outcome.

Evidence of a poor variation of movements at an early age can be found in the ground-breaking work of Phagava et al. (2008) who described an atypical pattern of General Movements (GMs) during the first 20 weeks of life in infants subsequently diagnosed with ASD. A significant number of infants with ASD show an abnormal quality of GMs (a “Poor Repertoire” of Writhing Movements) or even an absence of the age-specific “Fidgety Movements” pattern, suggesting that a reduced variation and an increased monotony in early spontaneous motor activity are likely to be observed in the first months of life in children with ASD.

As repetitiveness is a functional mechanism in the transition from spontaneous to voluntary movements and given that the frequency and the repertoire of repetitive movements have been proved to distinguish ASD from TD and DD infants from 12 months of life on, further study should be conducted on children prior to that time point. The purpose of this study is to verify if a higher frequency of repetitive movements (described through their rate and duration) could differentiate infants with ASD from infants with DD and TD, analyzing the age range between 6 and 12 months. In addition, our purpose is to describe the repertoire of these movements analyzing both the body part involved and their symmetry (i.e., if they are performed with both limbs simultaneously or with one limb).

## Materials and Methods

### Participants

We analyzed clips from home videos recorded by parents when their children were aged between 6 and 12 months. The sample was composed of 30 children, belonging to three different groups: 10 infants with ASD, 10 with DD, and 10 with TD.

Children with ASD and with DD were diagnosed at an age ranged between 3 and 4 years, respectively, with “ASD” or “Intellectual Disability” by expert child psychiatrists using DSM-5 criteria. Children were administered ADOS-2, ADI-R, the Griffiths Mental Developmental Scale-ER, and the Vineland Adaptive Behavior Scale-II in order to evaluate autistic symptomatology and intellectual functioning. At the time of diagnosis parents were asked to provide their home videos. Children belonging to the TD group were recruited from a local kindergarten at an age between 3 and 4 years. At the time of recruitment, parents were asked to provide their home videos. In this population, atypical development was excluded using the Child Behavior Checklist (Achenbach and Rescorla, 2000) (See **Table 1**). None of the children enrolled in this study showed hearing or visual impairments, associated neurological disorders or genetic syndromes.

**Table 1:** Description of the groups.

	ASD	TD	DD
*n*	10	10	10
M/F	8/2	5/5	7/3
Diagnosis	ADOS-G ADI-R	CBCL	ADOS-G ADI-R
	GMDS-ER (mean GQ:		GMDS-ER (mean GQ:
Cognitive development	59.26, SD: 8.49)	–	56.82, SD: 8.16)
Total number of videoclips	50	50	48
6–7 months	8	9	8
7–8 months	8	8	8
8–9 months	9	8	8
9–10 months	8	8	8
10–11 months	9	8	8
11–12 months	8	9	8
Total (mean) duration of			
videoclip (*in seconds*)	5980 (119.6)	6020 (120.4)	5955 (124.9)
6–7 months	880 (110)	950 (105.5)	995 (124.4)
7–8 months	1020 (127.5)	1005 (125.6)	940 (117.5)
8–9 months	1030 (114.4)	1060 (132.5)	1005 (125.6)
9–10 months	1100 (137.5)	1055 (131.9)	960 (120)
10–11 months	900 (100)	950 (118.7)	1010 (126.2)
11–12 months	1050 (131.25)	1000 (111.1)	1045 (130.6)

The study was carried out in accordance with the recommendations of the Scientific Institute “Stella Maris Foundation” institutional review board. Parents signed an Informed Consent Form, in accordance with the Declaration of Helsinki, authorizing the storage and study of their video material. Copies of the videos were made and coded with an identification number to preserve confidentiality and anonymity.

### Video Selection and Editing Procedures

Video-clips of the age-range of 6–12 months were selected from home videos of thirty children (10 ASD, 10 TD, and 10 DD). We first extracted all the sequences in which the entire body of the child was clearly visible. Sequences were cropped in order to obtain an equal distribution, between groups, of the position assumed by the infant during the sequences and an equal number of scenes with the presence of objects and caregiver or another family member. A total of 148 video-clips lasting at least 1 minute and a total of 10 min (±20”) of video-clips for each subject was finally available for coding (See **Table 1**). Then, an ANOVA was performed to verify the comparability of the selected video-clips in terms of mean duration for each position (see Supplementary Material 1). A research assistant blind to the children’s diagnosis performed the selection.

### Measures

We developed a grid for coding Repetitive Movement Episodes (RMEs) considering head, mouth and tongue, arms, hands, lower limbs and trunk as part of the infant’s body potentially involved in repetitive movements. We defined as RMEs the repetition for at least two times consecutively and in a very restricted time frame (1 s) of an identical movement pattern (flexion, extension, rotation, abduction, adduction or elevation in all possible directions).

The grid (see **Table 2**) consisted of a total of seven items describing the part of body involved. Some items (arms, lower limbs, hands, and fingers) had a modifier to specify if the movement was bilateral or unilateral. For these items, total scores combining unilateral and bilateral movements were also taken into account.

**Table 2:** Description of the Repetitive Movement Episodes (RMEs).

Item	Description	Modifiers
Head	Repeated pattern of movements of neck and head in all possible directions.	–
Mouth and		
Tongue	Repeated oral movements without the presence of objects or body parts that approach or touch the mouth.	–
Arms	Repeated pattern of movements of the arms starting from shoulder or elbow.	Unilateral Bilateral Total
Hands	Repeated pattern of movements of the hands starting from wrist, and without distal manipulation pattern with objects or body parts.	Unilateral Bilateral Total
Fingers	Repeated pattern of movements of the fingers starting from phalanx, and without distal manipulation pattern with objects or body parts.	Unilateral Bilateral Total
Trunk	Repeated pattern of movements of trunk in all possible directions.	–
Lower limbs	Repeated pattern of movements of legs or/and feet starting from hip, knee or ankle, and without distal manipulation pattern with objects or body parts.	Unilateral Bilateral Total

### Video Coding Procedures

Repetitive movement episodes were coded by two undergraduate developmental therapists blind to the research hypothesis and to the children’s diagnosis, using a computer-based coding system, the Observer XT 10.0 (Noldus, 2010), professional software for the storing, management and analysis of observational data.

The two coders were trained to use the software and to detect the RMEs, observing and coding videos of children with ASD, DD, and TD not included in the sample. A preliminary agreement (Cohen’s Kappa ≥ 0.70) with an expert developmental therapist (GP) and between the two coders was obtained on five matched unselected video-clips for each group (tolerance window was set at 2 s).

The selected video-clips were randomly assigned to the two coders: each coder coded 50% (*n* = 74) of the video-clips, plus 25% (*n* = 19) of the video-clips assigned to the other coder. A satisfactory agreement (Cohen’s Kappa = 0.85) was further obtained at the end of the coding activities on the video-clips coded by both coders (25% of the total video-clips, *n* = 38). Videos with a high disagreement were coded by the expert developmental therapist, blind to group membership, and then included in the sample.

### Data Analysis

This study was carried out with a similar methodology to that used in previous studies (Muratori et al., 2011; Apicella et al., 2013): the rate of RMEs per minute (frequency) and the percentage of the RMEs duration compared to the total length of the video-clips (duration) was calculated for each item. Rate per minute and percentage duration are proved to be a useful measure for the occurrence and the prevalence of target behaviors within observations with a variable duration.

For each parameter, three comparisons among subgroups (ASD vs. TD, ASD vs. DD, and TD vs. DD) were carried out with the Mann-Whitney *U* test. An alpha significance level of 0.05 was chosen. Bonferroni’s correction was adopted to adjust for multiple comparisons. Adjustment took into account for number of comparisons among groups (ASD vs. TD, ASD vs. DD, and TD vs. DD: three comparisons) which we planned when designed the study, but – due to the explorative nature of our study – adjustment did not account for number of parameters. Therefore, the corrected alpha level to judge statistical significance was 0.017. The effectiveness of each parameter in discriminating between groups was assessed by the Receiver Operating Characteristic (ROC) curve analyses. Non-parametric ROC curves analyzed ASD vs. TD, ASD vs. DD, and TD vs. DD.

The area under the ROC curve (AUC) was calculated as a measure of diagnostic accuracy. Cut points with fixed sensitivity were obtained for accurate (AUC ≥ 0.80) parameters from analysis of the ROC curve. When AUC was low (≤0.20) ROC analysis was carried out by changing the reference group to obtain the appropriate estimate of the discriminatory power of the parameter. The level of significance was 0.05 and Bonferroni’s correction was adopted to control for type I error in multiple comparisons. Statistical analyses were carried out with STATA 13 (StataCorp, 2013).

## Results

### Comparisons between Groups

See Supplementary Materials 1 and 2 for summary statistics for each item by group and for *p*-values of statistically significant comparisons.

**FIGURE 1:**
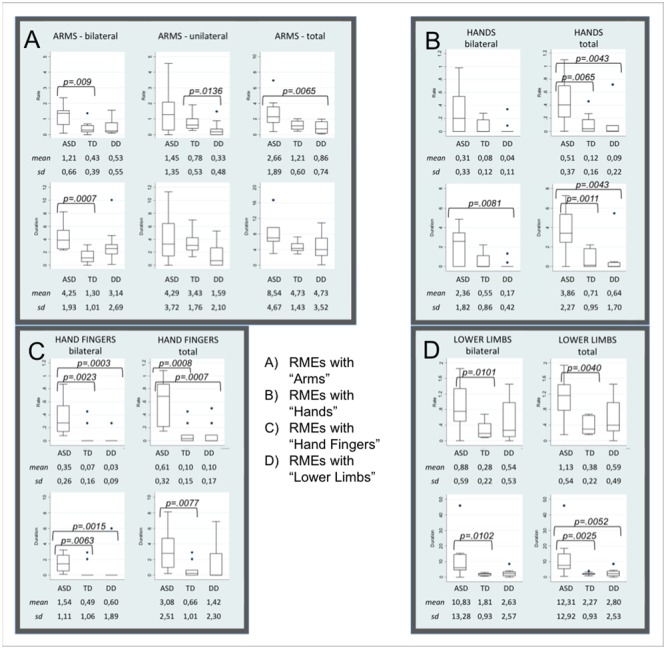
Box Plots of items distribution by groups. Box Plots show the distribution of each parameter in Autism Spectrum Disorders (ASD), Typical Development (TD), and Developmental Delay (DD) subjects. **(A–D)** Shown mean and standard deviation of the rate and the duration of RMEs with Arms (bilateral, unilateral, and total, **A**), Hands (bilateral and total, **B**), Fingers (bilateral and total, **C**), Lower Limbs (bilateral and total, **D**). The line in the middle of the box represents the median. The box extends from the 25th percentile (x[25]) to the 75th percentile (x[75]), the so-called interquartile range (IQ). The lines emerging from the box extend to the upper and lower adjacent values. The upper adjacent value is defined as the largest data point less than or equal to x[75] + 1.5 IQ. The lower adjacent value is the smallest data point greater than or equal to x[25] – 1.5 IQ. Dots indicate observed data points more extreme than the adjacent values (referred to as outliers).

*P* values of the Mann-Whitney test between groups (see Supplementary Material 3) revealed that frequencies and durations of RMEs with arms, hands, fingers, and lower limbs were significantly higher in ASD than in the other two groups. Distributions in the three groups, medians, interquartile ranges, means, standard deviation and *p*-values of each statistically significant item are showed in the Box Plots (**Figure 1**).

With respect to the TD group, infants with ASD show significantly higher frequency and duration of the following RMEs: Arms-bilateral, Hands total, Fingers bilateral and total, Lower limbs bilateral and total. Significantly higher frequency or duration of the following RMEs (Arms total, Hands bilateral and total, Fingers bilateral and total, Lower limbs bilateral and total) were observed in infants with ASD compared with infants with DD. Box Plots in **Figures 1B,C** show that TD and DD infants have a median of 0 in the frequency and in the duration of RMEs of hands and fingers (both bilateral and unilateral).

Moreover, the comparison between TD and DD groups revealed that frequency and duration of RMEs with Arms-unilateral was significantly higher in the TD group.

When comparisons among groups were carried out by including only male subjects, differences and statistical significance remained. Sometimes *p*-value missed statistical significance when accounting for multiple comparisons, but it was expected as the sample size was smaller (ASD = 9; TD = 7; DD = 6) (Data not shown).

### Item Discrimination Effectiveness (see **Table 3**)

**Table 3:** Diagnostic accuracy (AUC, area under the ROC curve) and definition of cutpoints for parameters significantly different between groups for sensitivity and specificity (gray cells refer to comparisons which do not achieve statistical significance).

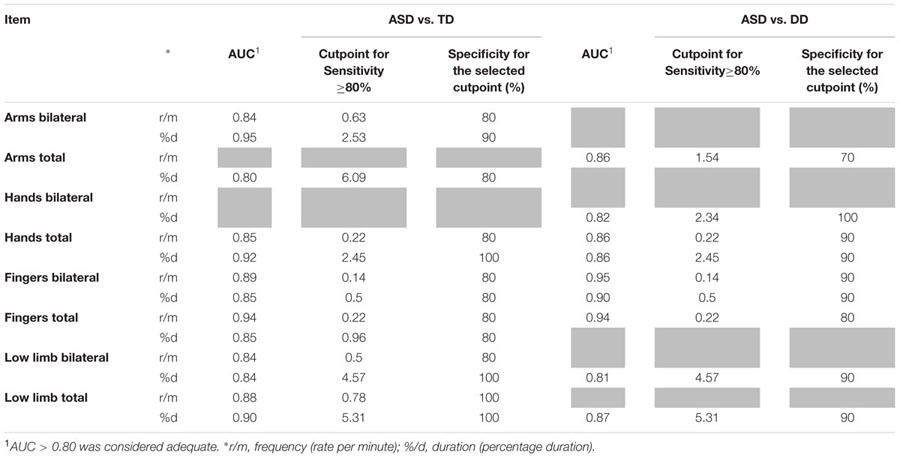

Receiver operating Characteristic curves were used to assess the diagnostic accuracy of those items that significantly discriminated ASD from the other two groups. An AUC ≥ 0.80 was considered an adequate discriminatory power. All RMEs items that showed significant differences between groups also showed an adequate diagnostic accuracy (AUC ≥ 80%). For these items, cut points corresponding to sensitivity ≥ 80% were determined with the aim of defining potential diagnostic criteria. Specificity corresponding to the selected cut points was high (≥70%).

## Discussions

The aim of this study was to investigate the role of repetitive movements in distinguishing between infants who develop ASD from those who do not. To achieve this aim we conducted a retrospective study to compare the frequency and the duration of a large variety of RMEs in infants with typical and atypical development during the second semester of life.

With respect to the frequency of RMEs, we found that significantly higher total scores in RMEs with arms, hands, fingers and lower limbs were able to distinguish ASD infants from both DD and TD infants. For these items, a satisfactory diagnostic efficiency (AUC ranging between 0.84 and 0.94, see **Table 3**) was also found. The respective cut points, selected if the sensitivity was ≥ 80%, show specificity values between 80 and 100% (**Table 3**). When distinguishing between unilateral and bilateral RMEs we found no significant difference between the distributions of unilateral RMEs in ASD and DD/TD with the exception of frequency and duration of unilateral RMEs with arms, which reliably distinguish between TD infants and DD infants. Taken together, these two observations might suggest that unilateral, rather than bilateral, repetitive movements are present in typical motor development and pave the way for the emergence of voluntary movement (Thelen, 1979).

Hence, we described a repertoire of repetitive movements in children with ASD composed mainly of bilateral RMEs with arms, hands, fingers and lower limbs. As we did not find any differences in these variables between TD and DD infants, we might describe patterns of more frequent and more persistent bilateral repetitive movements as a specific expression of the ASD condition.

Our results, taken together with our previous results showing the prevalence in ASD of a poor repertoire of GMs occurring during the first six months of life (Phagava et al., 2008), describe a lack of variation (as defined in the introduction) that characterizes the motor repertoire of very young infants with ASD. These early atypical motor trajectories could determine the lack of variability in finalized movements described in the second year of life (Wetherby et al., 2004; Loh et al., 2007; Elison et al., 2014).

The transition from the early abnormal quality of the newborn’s spontaneous motor activity to the specific patterns of bilateral repetitive movements and, subsequently, to motor stereotypes could be hypothesized as a sort of poor variation “gone astray.” In other words, motor repetitiveness is not limited to the appropriate age, whereas more finalized and voluntary movements fail to appear when they should. In accordance with Teitelbaum’s study, describing the permanence of infantile reflexes beyond the period of their physiological presence (“reflexes gone astray”) (Teitelbaum et al., 2004), we can hypothesize a continuum between the poor spontaneous motor activity and the reduced variation observed in the first months of life in ASD and the repetitive movements observed in the second half of the first year of life.

Considering the importance of the evolutionary balance between motor repetitiveness and variation, it can be hypothesized that an increase in repetitiveness, to the detriment of variation, could play a role in the motor delay observed initially in the first and second year of life (Ozonoff et al., 2008; Libertus et al., 2014) and then later in the third and fourth year of life (Provost et al., 2007). On the other hand, the high frequency and duration of repetitive movements with hands and fingers could suggest an atypical use of hand movements which, at this age, as in a certain period of human phylogenetic development, typically start to be used to gesture and communicate with others (Camaioni, 1997; Corballis, 2002). Thus, the presence of repetitive movements might reduce the possibility for a communicative use of hands. This hypothesis might be in line with evidence from the literature that suggests a correlation between early motor impairments and later communication delays (Bhat et al., 2012).

## Conclusion

The authors are fully aware of two main limitations of the present study. The first limit is inherent to the study design based on the retrospective analysis of naïve and not fully standardized video material and has been widely discussed in the literature concerning the retrospective use of home videos as a mitigating factor of reproducibility of the results (Costanzo et al., 2015). Indeed, the unavailability of a recording date for all videos made it impossible to know the exact age (weeks and days) of all the infants. However, the reduced possibility to standardize the video sequences subjected to analysis is in part balanced by the fully natural condition in which the videos are recorded. The second limit consists in the limited sample size, making our results absolutely preliminary. If on the one hand, we are inclined to consider very carefully the presence of a bilateral pattern of repetitive movements as a specific and sensitive behavioral marker in children with ASD during the first year of life, on the other hand, we think that the insights from this line of research are very promising and deserve to be examined in depth, with studies which use more stable and replicable methodologies, such as prospective designs. To our knowledge, this is the first study on the variation of infant’s motor repertoire at this early age. We believe that the limitations presented by its small sample size and retrospective designs are outweighed by its originality and thorough video-based analysis.

The present data showed higher frequency and duration of RMEs at the early age of 6 to 12 months in infants subsequently diagnosed with ASD compared to TD and DD infants. Our results indicate the presence, at this age, of specific patterns of repetitive movements, mainly composed of bilateral movements with arms, lower limbs, hands, and fingers. During this temporal window, these specific motor patterns emerge and remain stable over time, achieving a satisfactory diagnostic significance. In particular, repetitive movements with hands and fingers could represent highly sensitive target behaviors, not only in the administration of the ADOS-2 Toddler Module (Luyster et al., 2009) but also in early risk screening instruments, such as the AOSI (Bryson et al., 2008).

Finally, greater attention should be given to repetitive movements as specific risk markers for an early diagnosis.

Despite being aware that the so-called “Poor Repertoire of GMs” is a non-specific marker and has a low predictive power (Einspieler and Prechtl, 2005), especially when considered alone, we believe that the evaluation of GMs in the first weeks of life, together with the subsequent assessment of repetitive movements during the second half of the first year, should be further implemented in prospective studies through a more rigorous methodology, in order to verify the hypothesis of a continuum between poor repertoire and motor repetitiveness, and to clarify their potential as very early “motor markers” of ASD.

## Author Contributions

GP, VC, NC, MP, MS, FM, and FA participated in the design, execution and analysis of the paper by GP and colleagues, entitled “Bilateral patterns of repetitive movements in 6- to 12-month-old infants with Autism Spectrum Disorders” and declare they have seen and approved the final version and that it has neither been published nor submitted elsewhere.

## Conflict of Interest Statement

The authors declare that the research was conducted in the absence of any commercial or financial relationships that could be construed as a potential conflict of interest.
