# Supplementary material for: Bilateral Patterns of Repetitive Movements in 6- to 12-Month-Old Infants with Autism Spectrum Disorders
Source: Front Psychol. 2017 Jul 11;8:1168. doi: 10.3389/fpsyg.2017.01168 (PMC5504227; doi:10.3389/fpsyg.2017.01168)
Supplement: Supplementary file 1 [file Supplementary_Material.DOCX]

**Supplemental Material 1. Preliminary analysis**

| **Infant’s posture** | **Group** | **Mean duration (s.d.)** | **One-Way Anova** | |
| --- | --- | --- | --- | --- |
|  |  |  | *F* | *p-value* |
| **Prone** | ASD | 0,2 (0,42) | 0,29 | 0,75 |
|  | DD | 0,09 (0,30) |  |  |
|  | TD | 0,2 (0,42) |  |  |
| **Supine** | ASD | 0,6 (0,84) | 2,27 | 0,12 |
|  | DD | 0,18 (0,40) |  |  |
|  | TD | 0,1 (0,32) |  |  |
| **Seated** | ASD | 2,2 (0,91) | 1,63 | 0,21 |
|  | DD | 2,90 (2,11) |  |  |
|  | TD | 3,7 (2,21) |  |  |
| **Erect** | ASD | 0,5 (0,53) | 0,91 | 0,41 |
|  | DD | 0,18 (0,40) |  |  |
|  | TD | 0,41 (0,69) |  |  |
| **Mixed (multiple changes in the posture)** | ASD | 0,9 (0,9) | 0,64 | 0,53 |
|  | DD | 1,36 (0,92) |  |  |
|  | TD | 1 (1,05) |  |  |
| **Object presence** | ASD | 2,7 (1,05) | 2,84 | 0,11 |
|  | DD | 2,81 (1,40) |  |  |
|  | TD | 4,2 (2,09) |  |  |
| **Caregiver presence** | ASD | 2 (094) | 3,34 | 0,45 |
|  | DD | 3,36 (1,02) |  |  |
|  | TD | 3,6 (2,22) |  |  |
| **Other children/familiar presence** | ASD | 0,7 (1,05) | 0,71 | 0,50 |
|  | DD | 1,45 (2,58) |  |  |
|  | TD | 0,6 (1,26) |  |  |

***Insert as supplemental material***

**Supplemental Material 2: Summary statistics for each parameter by group**

**[mean, standard deviation (sd), median, interquartile range (iqr)]**

|  |  | **ASD** | | | | **TD** | | | | **DD** | | | |
| --- | --- | --- | --- | --- | --- | --- | --- | --- | --- | --- | --- | --- | --- |
| **Items *** | | *mean* | *sd* | *median* | *iqr* | *mean* | *sd* | *median* | *iqr* | *mean* | *sd* | *median* | *iqr* |
| Head | r/m | 0.44 | 0.73 | 0.05 | 0.65 | 0.23 | 0.45 | 0.00 | 0.25 | 0.25 | 0.73 | 0.00 | 0.09 |
| Head | %d | 0.80 | 1.33 | 0.35 | 0.98 | 0.89 | 1.59 | 0.00 | 0.84 | 0.23 | 0.41 | 0.00 | 0.50 |
| Mouth | r/m | 0.09 | 0.19 | 0.00 | 0.07 | 0.11 | 0.17 | 0.04 | 0.09 | 0.09 | 0.15 | 0.00 | 0.09 |
| Mouth | %d | 0.73 | 1.45 | 0.00 | 1.12 | 0.94 | 1.45 | 0.10 | 1.93 | 1.07 | 1.94 | 0.00 | 1.58 |
| Arm Unil | r/m | 1.45 | 1.35 | 1.28 | 1.80 | 0.78 | 0.53 | 0.62 | 0.61 | 0.33 | 0.48 | 0.18 | 0.36 |
| Arm Unil | %d | 4.29 | 3.72 | 3.26 | 5.03 | 3.43 | 1.76 | 3.04 | 1.87 | 1.59 | 2.10 | 0.70 | 2.67 |
| Arm Bil | r/m | 1.21 | 0.66 | 1.36 | 0.91 | 0.43 | 0.39 | 0.30 | 0.41 | 0.53 | 0.55 | 0.24 | 0.60 |
| Arm Bil | %d | 4.25 | 1.93 | 3.87 | 2.86 | 1.30 | 1.01 | 1.14 | 1.62 | 3.14 | 2.69 | 2.57 | 1.43 |
| Arm Tot | r/m | 2.66 | 1.89 | 2.28 | 2.02 | 1.21 | 0.60 | 1.12 | 1.05 | 0.86 | 0.74 | 0.74 | 1.47 |
| Arm Tot | %d | 8.54 | 4.67 | 7.02 | 3.61 | 4.73 | 1.43 | 4.34 | 1.81 | 4.73 | 3.52 | 4.01 | 4.53 |
| Hand Unil | r/m | 0.20 | 0.25 | 0.05 | 0.45 | 0.04 | 0.06 | 0.00 | 0.08 | 0.05 | 0.12 | 0.00 | 0.00 |
| Hand Unil | %d | 1.50 | 1.85 | 0.41 | 3.56 | 0.16 | 0.31 | 0.00 | 0.25 | 0.46 | 1.30 | 0.00 | 0.00 |
| Hand Bil | r/m | 0.31 | 0.33 | 0.21 | 0.54 | 0.08 | 0.12 | 0.00 | 0.18 | 0.04 | 0.11 | 0.00 | 0.00 |
| Hand Bil | %d | 2.36 | 1.82 | 2.58 | 3.45 | 0.55 | 0.86 | 0.00 | 1.12 | 0.17 | 0.42 | 0.00 | 0.00 |
| Hand Tot | r/m | 0.51 | 0.37 | 0.41 | 0.48 | 0.12 | 0.16 | 0.04 | 0.18 | 0.09 | 0.22 | 0.00 | 0.09 |
| Hand Tot | %d | 3.86 | 2.27 | 3.42 | 2.92 | 0.71 | 0.95 | 0.13 | 1.83 | 0.64 | 1.70 | 0.00 | 0.41 |
| Finger Unil | r/m | 0.26 | 0.28 | 0.11 | 0.54 | 0.03 | 0.04 | 0.00 | 0.07 | 0.07 | 0.16 | 0.00 | 0.09 |
| Finger Unil | %d | 1.54 | 2.19 | 0.68 | 1.50 | 0.16 | 0.27 | 0.00 | 0.44 | 0.82 | 1.26 | 0.00 | 1.16 |
| Finger Bil | r/m | 0.35 | 0.26 | 0.28 | 0.40 | 0.07 | 0.16 | 0.00 | 0.00 | 0.03 | 0.09 | 0.00 | 0.00 |
| Finger Bil | %d | 1.54 | 1.11 | 1.46 | 2.06 | 0.49 | 1.06 | 0.00 | 0.00 | 0.60 | 1.89 | 0.00 | 0.00 |
| Finger Tot | r/m | 0.61 | 0.32 | 0.69 | 0.65 | 0.10 | 0.15 | 0.04 | 0.09 | 0.10 | 0.17 | 0.00 | 0.09 |
| Finger Tot | %d | 3.08 | 2.51 | 2.80 | 3.76 | 0.66 | 1.01 | 0.22 | 0.64 | 1.42 | 2.30 | 0.00 | 2.76 |
| Trunk | r/m | 0.29 | 0.28 | 0.32 | 0.43 | 0.17 | 0.22 | 0.07 | 0.27 | 0.13 | 0.16 | 0.09 | 0.18 |
| Trunk | %d | 2.15 | 2.19 | 2.13 | 3.45 | 0.50 | 0.65 | 0.24 | 1.04 | 0.63 | 0.88 | 0.25 | 1.17 |
| LowLimb Unil | r/m | 0.25 | 0.29 | 0.15 | 0.38 | 0.10 | 0.10 | 0.09 | 0.13 | 0.05 | 0.09 | 0.00 | 0.09 |
| LowLimb Unil | %d | 1.48 | 1.88 | 0.61 | 2.96 | 0.46 | 0.49 | 0.23 | 1.06 | 0.17 | 0.29 | 0.00 | 0.39 |
| LowLimb Bil | r/m | 0.88 | 0.59 | 0.76 | 0.84 | 0.28 | 0.22 | 0.19 | 0.35 | 0.54 | 0.53 | 0.27 | 0.88 |
| LowLimb Bil | %d | 10.83 | 13.28 | 6.16 | 10.03 | 1.81 | 0.93 | 2.00 | 1.43 | 2.63 | 2.57 | 2.31 | 2.48 |
| LowLimb Tot | r/m | 1.13 | 0.54 | 1.16 | 0.66 | 0.38 | 0.22 | 0.30 | 0.47 | 0.59 | 0.49 | 0.40 | 0.73 |
| LowLimb Tot | %d | 12.31 | 12.92 | 7.68 | 9.69 | 2.27 | 0.93 | 2.33 | 0.75 | 2.80 | 2.53 | 2.31 | 2.88 |
|  | | | | | | | | | | | | | |

* r/m = frequency (rate per minute); %/d = duration (percentage duration)

***Insert as supplemental material***

**Supplemental Material 3: P-values of Mann-Whitney test of the comparisons between groups (grey cells refer to comparison which do not reach statistical significance)**

| **Items** |  | **p-value (Mann-Whitney)^1^** | | |
| --- | --- | --- | --- | --- |
|  | ***** | **ASD vs TD** | **DD vs TD** | **ASD vs DD** |
| **Arms Unil** | r/m |  | 0.0136 |  |
|  | %d |  |  |  |
| **Arms Bil** | r/m | 0.0090 |  |  |
|  | %d | 0.0007 |  |  |
| **Arms Tot** | r/m |  |  | 0.0065 |
|  | %d |  |  |  |
| **Hands Bil** | r/m |  |  |  |
|  | %d |  |  | 0.0081 |
| **Hands Tot** | r/m | 0.0065 |  | 0.0043 |
|  | %d | 0.0011 |  | 0.0043 |
| **Fingers Bil** | r/m | 0.0023 |  | 0.0003 |
|  | %d | 0.0063 |  | 0.0015 |
| **Fingers Tot** | r/m | 0.0008 |  | 0.0007 |
|  | %d | 0.0077 |  |  |
| **LowLimb Bil** | r/m | 0.0101 |  |  |
|  | %d | 0.0102 |  |  |
| **LowLimb Tot** | r/m | 0.0040 |  |  |
|  | %d | 0.0025 |  | 0.0052 |

**^1^** By adopting Bonferroni’s correction with 3 comparisons at alpha of 0.05, comparisons are significant if p<0.0167

* r/m = frequency (rate per minute); %/d = duration (percentage duration)

***Insert as supplemental material***
